# LGBTQ+ Inclusion and Support: An Analysis of Challenges and Opportunities Within 4-H

**DOI:** 10.5195/jyd.2021.1072

**Published:** 2021

**Authors:** Joseph J. Rand, Megan S. Paceley, Jessica N. Fish, Sloan Okrey Anderson

**Affiliations:** Extension Center for Youth Development, University of Minnesota; School of Social Welfare, University of Kansas; Department of Family Science, University of Maryland; Department of Family Social Science, University of Minnesota

**Keywords:** LGBTQ+, 4-H, inclusion, diversity, gay, lesbian, bisexual, trans, youth

## Abstract

LGBTQ+ youth experience health disparities compared with heterosexual and cisgender youth. Community-based, positive youth development organizations are an important resource to support and affirm LGBTQ+ youth. This study aimed to identify the opportunities and challenges in supporting LGBTQ+ youth within 4-H. The study took place in one state in the United States within a 4-H program and employed qualitative, community-based methods using SWOT (strengths, weaknesses, opportunities, threats) analyses and focus groups of 4-H staff, support staff, volunteers, and youth participants. The majority of participants were White and middle class with direct connections to the 4-H program. Thematic analyses were conducted by multiple analysts until consensus was reached. Challenges and opportunities emerged in 3 themes: (a) organizational climate; (b) policies and procedures; and (c) training, education, and resources. Two additional themes included opportunities only: (a) community engagement and (b) youth-specific resources. This study has important implications for the 4-H program, rural community practice, and research, including strategies to improve LGBTQ+ inclusivity through education, programs and policies, hiring, and community partnerships. Additionally, this study highlights the opportunity and unique positionality of the 4-H program to amplify youth voices in the creation of youth-specific resources.

Lesbian, gay, bisexual, transgender, queer, and questioning (LGBTQ+) youth are both a resilient and uniquely vulnerable population (Fish et al., 2020; Russell & Fish, 2016). Compared to heterosexual and cisgender youth, LGBTQ+ youth experience poorer mental health (Marshal et al., 2011; Russell & Fish, 2016) and greater substance use (Marshal et al., 2008; Mereish, 2019), including binge drinking, illicit drug use, and polysubstance use (Fish & Baams, 2018; Mereish, 2019). Additionally, LGBTQ+ youth are significantly more likely to attempt suicide compared with non-LGBTQ+ youth (Marshal et al., 2011; Perez-Brumer et al., 2017). As evidence of these disparities mounts, local and national stakeholders have taken interest in developing ways to support LGBTQ+ youth (Center for Disease Control, 2018; Office of Disease Prevention and Health Promotion, 2020; Substance Abuse and Mental Health Services Administration, 2020).

A growing body of science illustrates that sexual orientation and gender identity-related health disparities are a function of stigma, discrimination, bullying, and victimization (Goldbach et al., 2014; Hatzenbuehler, 2009). LGBTQ+ youth are more likely to report harassment, discrimination, and bullying in school contexts than heterosexual and cisgender youth (Toomey et al., 2018) and are susceptible to family rejection based on their sexuality or gender (Ryan et al., 2009). Additionally, when LGBTQ+ youth experience hostility based on their sexuality or gender within their community they have an increased risk of suicide (Hatzenbuehler & Keyes, 2013). These experiences with stigma and rejection help explain the differences between non-LGBTQ+ and LGBTQ+ youth mental health (Day et al., 2017; Goldbach et al., 2014; Perez-Brumer et al., 2017; Russell & Fish, 2016). Conversely, family acceptance and LGBTQ+-specific school protections are associated with better mental health and less substance use among LGBTQ+ youth (Hatzenbuehler, 2009; Ryan et al., 2010; Snapp et al., 2015). This suggests that affirming and accepting environments and interpersonal networks—perhaps not surprisingly—are a necessary support for positive youth development for this population and may foster resilience in the face of adversity (e.g., Toomey et al., 2018). Relatedly, when LGBTQ+ youth experience support in their communities, such as via supportive religious institutions, their mental and behavioral health improves (Hatzenbuehler, 2009).

One aspect of community that warrants critical empirical attention is that of community-based positive youth development organizations serving broad populations of youth and their ability to support LGBTQ+ youth. For example, 4-H programs can be a great source of support for youth, yet they may also have opportunities for growth in their ability to support the many LGBTQ+ youth and staff who engage with this program across the United States. Given the importance of community support for the well-being of LGBTQ+ youth, this study aimed to identify the challenges to LGBTQ+ inclusion and the opportunities to promote LGBTQ+ affirmation and support within 4-H. Findings from this study have the potential for important implications for both 4-H and other community-based organizations.

## LGBTQ+ Youth Development and Wellbeing

Even with great strides in LGBTQ+ visibility and rights, contemporary LGBTQ+ young people continue to navigate social and cultural contexts that are fundamentally rooted in and reify hetero- and cis-normativity. With each new release of the Youth Risk Behavioral Surveillance Survey (YRBS), findings continue to highlight the profound differences in mental health, substance use, and victimization between heterosexual, cisgender youth and their LGBTQ+ peers (Johns et al., 2018, 2019). More recent studies tracking trends in sexual orientation-related disparities in mental health, substance use, victimization, and family support highlight that these inequities have not subsided alongside the growing acceptance of LGBTQ+ people (Fish et al., 2019; Fish & Baams, 2018; Poteat et al., 2019; Raifman et al., 2020; Watson et al., 2019). Unfortunately, with the long-standing exclusion of gender identity measures in population-based data, these trend analyses have not been possible for transgender youth. Still, data on the health of transgender youth show similar, if not elevated, rates of poor mental and behavioral health relative to both cisgender heterosexual and sexual minority peers (Day et al., 2017; Perez-Brumer et al., 2017).

With increasing visibility and protections for LGBTQ+ people, many are left wondering why LGBTQ+ youth mental health remains urgent. Recent writings illustrate how—alongside changing cultural contexts for LGBTQ+ people—sexual and gender minority youth are empowered to come out at younger ages than decades past (Bishop et al., 2020); however, this disclosure now occurs during a developmental period in which LGBTQ+ youth are particularly vulnerable to peer disapproval, victimization, and family ruptures (Russell & Fish, 2016; 2019). Contemporary LGBTQ+ youth are therefore able to understand and disclose sexual and gender identity at younger ages, but are left to navigate potentially unsupportive spaces with fewer resources. Thus, youth programs that are able to provide support to LGBTQ+ youth remain a critical and necessary area of future research and development.

### Positive Youth Development

One population that has yet to receive adequate attention in the application of positive youth development (PYD) is LGBTQ+ youth. PYD (Benson et al., 1998; Silbereisen & Lerner, 2007) guides much of the nation’s youth policy and programming initiatives (Benson et al., 2011; Catalano et al., 2004; Roth & Brooks-Gunn, 2016), including 4-H. PYD is rooted in prevention and is an approach to programming that seeks to promote youth social connection, socioemotional competence, self-determination, self-efficacy, future orientation, and prosocial behavior, among other prosocial outcomes across the various contexts in which youth develop, such as schools, family, and community (Catalano et al., 2004; Silbereisen & Lerner, 2007). Despite its widespread use in youth programming, PYD’s application to LGBTQ+ youth has been limited (Toomey et al., 2019). Preliminary research in this area has focused on the Developmental Assets Framework, which names 40 positive resources and strengths that youth need to thrive (Benson et al., 2011). Findings suggest that these assets operate similarly for heterosexual, cisgender, and LGBTQ+ youth (Syvertsen et al., 2019; Toomey et al., 2019). Perhaps not surprisingly, however, LGBTQ+ youth reported fewer internal (e.g., academic engagement, social competence) and external (e.g., support, belonging) assets than their heterosexual and cisgender peers (Syvertsen et al., 2019; Toomey et al., 2019); yet these assets would likely provide LGBTQ+ youth greater increased resilience and additional coping strategies in the face of adversity. With intentional efforts to enhance inclusion and affirmation of LGBTQ+ identities, community-based youth organizations, such as 4-H, may help to increase developmental assets among LGBTQ+ youth.

### LGBTQ+ Youth and Rural Community Context

Scholarship on LGBTQ+ youth often reflects and frames the experience of LGBTQ+ youth in urban contexts, a focus that ignores the geographic heterogeneity that often alters the experience of being an LGBTQ+ young person. Simultaneously, rural communities are often characterized as inherently hostile toward LGBTQ+ youth, with few opportunities for support or resources (Paceley, 2020). Indeed, some research suggests that LGBTQ+ youth in rural communities overhear more anti-LGBTQ+ sentiment (Kosciw et al., 2015) and have less access to supportive resources (Paceley, 2016; Paceley et al., 2019) than youth in urban communities. Alternatively, research indicates that rural LGBTQ+ youth are resilient and develop pathways to positive identity and well-being in ways that are different from urban youth. For example, Gray (2009) described how rural LGBTQ+ youth in Kentucky utilized non-LGBTQ+ spaces to form community, such as the local Walmart. Youth in her study also used online and social media outlets to communicate and find support across rural areas, a common strategy for rural LGBTQ+ youth (Gray, 2009; Paceley et al., in press).

Rather than position rural communities as inherently hostile, it may be useful to consider rural communities as promoting different strengths and values for LGBTQ+ youth and identify opportunities to strengthen these communities. Paceley (2020) described the necessity of understanding rural communities in light of their strengths rather than from a deficit-based approach. For example, rural communities may offer a sense of closeness and connection to others not found in urban spaces. Still, these studies remain rare in LGBTQ+ youth scholarship and more research is needed to identify the opportunities for support in rural communities for LGBTQ+ youth. One possibility is that existing youth organizations could provide support to LGBTQ+ youth, yet their impact and work with LGBTQ+ youth have not been explored. One such organization that is common in rural communities is 4-H.

## 4-H Programs: Historical Roots and Youth Engagement

4-H has its roots in the corn- and tomato-growing clubs of rural Clark County, Ohio in 1902. Realizing adults were often averse to changes in agricultural practices, researchers found that offering new strategies to young people often transferred to their parents, and “hands-on” learning in club settings arose from a need to connect public school education and country life. Membership in 4-H has seen remarkable growth throughout the 19^th^ and 20^th^ centuries. In 2018, the Annual Report by National 4-H indicated that 6 million youth were engaged in the program, with increasing participation among youth in metropolitan areas (National 4-H Council, 2018). For example, in 1975, only 36.7% of members reported living in a town of more than 10,000 compared to 56.7% in 2018 (National 4-H Council, n.d.). Today, the 4-H program focuses on providing meaningful opportunities to create sustainable community change through youth and adult relationships. This is accomplished through three primary content areas: civic engagement and leadership, healthy living, and science (CFERR, 2019). 4-H engages youth in programs that foster PYD with attention to the “5 C’s”: competence, confidence, connection, character, and caring (Lerner et al., 2013; Lerner et al., 2005).

Stated goals demonstrate National 4-H’s commitment to engage youth members and staff to accurately reflect the demographics of the United States by 2025 (CFERR, 2019). To support these efforts, unaffiliated groups like the 4-H Access, Equity, and Belonging Committee (AEBC) have formed. As part of the 4-H Program Leader’s Working Group the mission of the AEBC is to increase the capacity of the 4-H program to create an inclusive organizational culture (Program Leaders Working Group, n.d.). The committee is composed of champion groups focused on logic models, best practices, and strategies to attract marginalized groups of young people.

Existing champion groups focus on different groups of marginalized youth, including incarcerated youth, immigrant and refugee youth, LGBTQ+ youth, youth with mental health concerns, homeless youth, youth in foster care, disabled youth, youth in poverty, and racial and ethnic minoritized youth. Despite this stated commitment, 4-H has primarily focused on race, ethnicity, and socio-economic factors. While an LGBTQ+ champion group exists within the AEBC, National 4-H does not name gender identity or sexual orientation in its stated diversification goals.

Although 4-H does not collect data about LGBTQ+ identity, it does offer a document aimed at increased equity for LGBTQ+ participants (AEBC, 2020). While many 4-H clubs were co-ed, membership activities were created specific to gender based on young people’s interests. At present, the 4-H program offers gendered programming and measures participation using the gender binary. For example, 2014 participation was reported as 51.7% female and 48.3% male (National 4-H Council, 2014). As more LGBTQ+ youth engage in 4-H, many in the organization have recognized necessary changes to policies and programs. Still, attempts to make 4-H more welcoming and inclusive for LGBTQ+ youth are scattershot and largely tied to the willingness of local educators and staff to integrate these policies either on their own or through outside support groups, but without the backing of national 4-H support.

Given the relevance of 4-H to youth, many of whom are rural, and the need to identify supportive community-based resources, this study aims to understand the challenges and opportunities to supporting the positive development of LGBTQ+ youth within 4-H.

## Methods

Community-based case study methods were used to center the expertise of 4-H youth, staff, and volunteers in one state and provide benefits to both the organization and the research. As a 4-H affiliate and community member, the first author conducted a series of LGBTQ+ competency-building workshops with youth, staff, and volunteers affiliated with 4-H. Workshops were provided with the goal of engaging in a process of change and empowerment within the state’s 4-H program. Workshops included a SWOT (strengths, weaknesses, opportunities, threats) analysis of the 4-H program with an LGBTQ+ inclusion lens or a related structure and process using focused questions. A SWOT analysis is a process used to evaluate factors within and outside an organization that impact its functioning, effectiveness, or success (Helms, 2019). Strengths focus on what the organization is doing well and its capacities. Weaknesses include challenges faced by the organization, or areas in which they may lack resources or capabilities. Opportunities focus on the potential for growth and change from internal or external sources. Finally, threats include factors within or outside the organization that have the potential to damage or disrupt growth. SWOT analyses are often used in organizational planning and improvement and offer unique and innovative ways to collect qualitative methods, particularly within a single case study.

Participants were recruited through workshop descriptions provided in respective conference agendas (see Table 1). Although geographic data were not collected, these workshops engaged 4-H affiliates from diverse county backgrounds across the state, including large metropolitan cities and rural towns. Workshops were 45- to 90-minute sessions facilitated within larger conferences. A total of three separate SWOT analyses were conducted with different constituent groups (*n* = 68) within the state’s 4-H program (see Table 1). A fourth group with staff (*n* = 12), included specific questions used as prompts rather than just the SWOT headings as part of a competency-building workshop modified for support staff working in local county 4-H programs. The questions were designed to help develop supports and strategies to meet their specialized role, rather than a more general SWOT analysis. Question prompts included the following: *Where do you run into difficulty navigating LGBTQ+ related circumstances? What guidance do you need in your county to provide successful experiences for LGBTQ+ youth in your programs? What tools would be helpful in order to better navigate LGBTQ+ related circumstances? What opportunities do you see for 4-H to make our programs more inclusive for LGBTQ+ youth? Adults? Volunteers?*

Headings or questions were written on large Post-it notes and placed around the room. Participants worked in small groups rotating to each Post-it note to provide their thoughts and feedback. They were asked to place stars or check marks next to responses from previous groups with whom they agreed or whose sentiments resonated with them. Responses ranged from single words to phrases and questions. Once groups provided feedback on all Post-it notes, responses were discussed as a large group. Post-it notes were collected and data were compiled by the first author. Groups were not audio-recorded; however, the first author took notes as part of the data collection process. This research was approved by the University of Kansas Institutional Review Board.

### Data Analysis

Data were analyzed using thematic analysis (Braun & Clarke, 2006) by three coders. Thematic analysis is a flexible, yet rigorous method of qualitative analysis that allows for the discovery of patterns within a set of qualitative data. It is particularly useful with this data set given our goal of assessing patterns across constituent groups via SWOT and group meetings in which notes were taken, but without verbatim transcripts. It provides the structure to assess patterns without being theoretically driven. Our analytic process followed the steps laid out by Braun and Clarke (2006). The first author established familiarity with the data based on a review of the meeting notes, given he had been present at each meeting. Next, the second author and a student assistant read through all the notes to become familiar with the data without reviewing the first author’s notes. They independently created an initial set of codes; the second author combined their codes into an initial set of themes, discussing areas in which there were discrepancies. They compared their set of themes to the first author’s and, finding consistency, the second author used the set of themes to engage in multiple rounds of coding until near-final sets of themes and sub-themes were identified. After the themes were named and defined, a summary of findings was shared with the research team before compiling them for dissemination.

### Researcher Positionality

Given the qualitative and community-based nature of this study, it is important for us to reflect on our positionality within the research project. Author 1 is directly affiliated with 4-H through his position as youth development Extension faculty that includes research and advocacy for LGBTQ+ youth. He identifies as a White, gay, cisgender man that lives and works in rural communities. Author 2 is a social worker and scholar engaged in research with queer and trans youth and communities. She identifies as a White, queer, cisgender woman. Author 3 is a White, cisgender, queer woman with training in human development, family science, and couple and family therapy. Author 4 is a White, transgender social worker and scholar within the discipline of human development and family science. We engaged reflexively with these positionalities in terms of identities and what we bring to the research, as well as our professional roles and identities. As queer and/or trans scholars, social workers, practitioners, and youth development leaders, we take an explicit stance toward promoting equity and inclusion for LGBTQ+ youth; this lens was intentionally present in our data collection, analysis, and dissemination processes. Importantly, we also reflected on our positionality as White scholars and geographic positionality outside of the studied area for authors 2, 3, and 4.

## Findings

Findings include challenges specific to LGBTQ+ constituents within the 4-H program, as well as opportunities to address these challenges and promote safety and acceptance for 4-H LGBTQ+ youth. Challenges and opportunities were present in three themes: (a) organizational climate; (b) policies and procedures; and (c) training, education, and resources. Two additional themes included opportunities only: (a) community engagement and (b) youth-specific resources. The following sections illustrate these themes with a thick description of the findings based on meeting notes and the first author’s presence at the meetings. Table 2 summarizes these findings.

### Organizational Climate

The first theme encompasses the ways in which the organizational climate created challenges or opportunities to support LGBTQ+ youth in 4-H. We use Oswald et al.’s (2010) definition of climate as it relates to communities, given its connection to LGBTQ+ people, specifically. Therefore, we define climate as the level of support for or hostility toward LGBTQ+ people within the 4-H organization.

#### Challenges

Numerous factors contributed to a lack of LGBTQ+ support within the climate of 4-H. These factors were sometimes overt, such as experiencing or witnessing harassment and bullying based on real or perceived LGBTQ+ identity and a lack of intervention by adult leaders, staff, and volunteers. Group 4 shared instances of other youth misgendering, misnaming, and harassing LGBTQ+ youth or youth perceived to be LGBTQ+. This led LGBTQ+ youth to feel unsafe and unwelcome in the program. Feeling unsafe and unwelcome was exacerbated by a lack of intervention by program staff and leaders. Group 3 shared an experience in which an adult at a 4-H meeting responded to a discussion around creating inclusivity for LGBTQ+ youth by saying, “*Why do we want those people?*” They shared other examples of overt hostility by program staff and adults involved with the program, such as purposefully saying “*mom and dad*,” even after a youth shared that they had two moms.

There were also myriad subtle ways participants perceived the organizational climate creating challenges for the inclusion of LGBTQ+ youth. Groups 1 and 4 expressed that the lack of diversity pertaining to gender, sexuality, and other forms of diversity among staff, volunteers, and youth sent a message that LGBTQ+ youth were not welcome. The lack of diversity also made it challenging to create positive change within the organization as allies and advocates lacked diverse leaders in positions of power. Another way that diversity was challenged was by the assumption of heterosexuality in the ways in which staff or volunteers communicated. For example, some constituents suggested there were no LGBTQ+ youth in 4-H or assumed all youths’ dating partners were of a different gender.

Multiple groups discussed the challenges present in assuming a gender binary, such that youth who identified as non-binary were forced to “choose” a gender or participate in gendered activities based on their sex assigned at birth. This showed up in the ways in which activities, bathrooms, sleeping arrangements at camp, and other activities were organized into a sex assigned at birth binary of boys and girls.

The final subtle way in which organizational climate posed challenges was through the conservative religious tone conveyed by some individual members, even though 4-H is not a religious organization. One program staff shared how a chapter organized a service-learning project with a religiously based organization known for its anti-LGBTQ+ stance and how this created a barrier to participation for LGBTQ+ youth or youth with LGBTQ+ parents. Another staff member shared how an adult affiliated with 4-H challenged the need to support LGBTQ+ youth because it contradicted individual religious beliefs.

#### Opportunities

It is important to note that although participants across constituent groups within 4-H were critical of climate factors that resulted in hostility or challenges for LGBTQ+ youth, they also expressed positive sentiments about the climate of 4-H and the opportunities it held for creating inclusion and safety for LGBTQ+ youth. These opportunities were discussed in two ways: (a) features within the existing climate that can promote acceptance if they are specific to LGBTQ+ youth and (b) new opportunities to improve the climate for LGBTQ+ youth.

Within the existing climate of 4-H, constituents across all of the groups shared how the mission and values of 4-H are rooted in acceptance of and support for all youth—there are no exclusionary criteria. Youth and volunteers shared that 4-H promotes a sense of togetherness, positivity, and safety, as well as independence and leadership for all youth. Participants also indicated that although there are many religious members of 4-H in their local, often rural communities, 4-H is not affiliated with a particular religion, which provides opportunities to engage across religious beliefs and identities. Finally, youth were able to identify numerous accepting staff, volunteers, and leaders who actively support LGBTQ+ youth even when hostility or harassment were present.

There were also opportunities to shift the climate in new and positive directions in order to better support LGBTQ+ youth. Multiple groups identified concrete strategies that fit neatly within the existing climate opportunities. Youth, alumni, and volunteers suggested that increasing the diversity and visibility of LGBTQ+ youth, staff, volunteers, and leaders would create additional opportunities to support existing and future LGBTQ+ youth members. They recognized the importance of the visibility of LGBTQ+ identities in promoting a positive climate for LGBTQ+ youth, but also recognized that if other climate factors do not shift toward supportive opportunities, diversity and visibility will not be maintained or may result in safety concerns. Therefore, participants described the need for specific strategies to make visible a climate change for LGBTQ+ people. Some examples included adding pronoun options to nametags and providing group activity opportunities without gendering them. Other opportunities were related to policies and procedures or education and training and, thus, are discussed below.

### Policies & Procedures

The second theme encompassed the policies and procedures within 4-H (broadly and locally) that created challenges or opportunities to support LGBTQ+ youth. Policies and procedures are one aspect of climate (Oswald et al., 2010; Paceley et al., 2020) however, we separate them here to illustrate their importance in establishing an environment that supports or creates challenges to supporting LGBTQ+ youth.

#### Challenges

Policies and procedures were challenging when they limited acceptance for and inclusion of LGBTQ+ youth. Youth, alumni, and volunteers described how 4-H lacked policies that explicitly included LGBTQ+ youth, staff, and volunteers. Although their general policies were affirming of all youth broadly, participants shared that in the absence of that explicit language, members of the organization could elect to ignore LGBTQ+ issues or overtly discriminate.

Finally, the assumption that all youth are cisgender and the enforcement of gender as a binary, discussed in organizational climate, also showed up in policy challenges, specifically in terms of policies pertaining to group activities, bathrooms, and sleeping arrangements. Gender binary-based policies can be complex for non-binary youth to navigate and can reinforce stereotypes that create challenges for youth who are questioning their gender identity or identifying as a gender other than their sex assigned at birth.

#### Opportunities

Constituents remained positive in their perception of 4-H and noted several areas that were opportunities for 4-H policies and procedures to be more inclusive and affirming of LGBTQ+ youth. First, they suggested revision to the equal employment opportunity policy to include sexual orientation and gender identity as protected identity classes. Although this specific policy was named, participants also noted the need to explicitly apply policies related to acceptance, non-discrimination, and bullying to LGBTQ+ people. Another policy opportunity that would enhance support for LGBTQ+ youth, identified across constituent groups, was to change or remove policies that promote or enforce a gender binary. This may include policies such as dress codes, gender assignments, programs specifically designed for or marketed for one gender (e.g., girls/boys lock-in, camp, overnight retreats, etc.), or programs that reinforce gender stereotypes subversively (sewing and scrapbooking events geared toward girls, fishing and shooting sports geared toward boys). When discussing the need for policy change, program staff suggested that opportunities existed to collaborate and utilize the expertise from local universities. They identified university diversity offices and Extension units as potential resources, suggesting they could assist with policy efforts.

### Training, Education, and Resources

The third theme encompassed challenges and opportunities within 4-H training, education, and resources that prevented or could create a supportive environment for LGBTQ+ youth. Participants primarily discussed opportunities for positive growth within this theme.

#### Challenges

Program staff noted a stark lack of training and education resources to assist them in supporting LGBTQ+ youth and facilitating change within the organization. They shared that their volunteer training excluded content on LGBTQ+ youth and they lacked guidance on LGBTQ+-related language, how to create safe and affirming environments, and how to respond to criticism and resistance. All constituents shared how a lack of LGBTQ+-inclusive or supportive resources limited their ability to support LGBTQ+ youth. They specifically noted a lack of resources on responding to harassment and discrimination, addressing anti-LGBTQ+ sentiment in the community, and creating inclusive and affirming program materials.

#### Opportunities

In many ways, the opportunities noted by participants are the opposite of the challenges—ways in which 4-H could address the lack of training, education, and resources to support LGBTQ+ youth. However, participants were more specific than general calls for “additional training.” They identified specific opportunities to create inclusive training and educational resources. They indicated that opportunities existed to incorporate LGBTQ+ content into volunteer and camp counselor training, as well as within training for youth. Having LGBTQ+ content in the training would provide skills and resources for staff and volunteers, and also send a message that LGBTQ+ youth are supported. Program staff also suggested that training should be trauma-informed in order to address the stigma and victimization that LGBTQ+ youth experience. They suggested training should include a focus on LGBTQ+ language, creating safe environments, and strategies for communicating their inclusivity to all constituents. Some youth suggested a regular LGBTQ+ keynote speaker at large events.

Program staff specifically identified opportunities to enhance their resources to be more LGBTQ+ supportive. They identified an opportunity to update forms and documents to be more inclusive of various genders and parent/family structures. They also suggested adding publications and advertisements that explicitly include LGBTQ+ youth in 4-H, including LGBTQ+ youth stories and a video featuring inclusivity and visibility of LGBTQ+ identities. They also suggested a need for resources that promote visibility and support, such as safe space stickers. Youth, alumni, and volunteers suggested that a best practices handbook with housing, restroom, language, and mental health guidance would be an asset to program staff and volunteers.

### Community Engagement

The fourth theme included opportunities for 4-H chapters and the larger organization to engage with the community in ways that support LGBTQ+ youth. Program staff suggested determining where LGBTQ+ youth find support in the community and allying with those agencies or organizations. They indicated that a possible resource that could improve 4-H’s ability to engage with the community was the local university, specifically diversity and extension offices. These offices may be able to facilitate training and resources identified above, as well as assist with larger community-level events, such as LGBTQ+ Pride or other LGBTQ+ events. Youth participants echoed this desire to have 4-H involved in the local community via participation at Pride events or connecting with other LGBTQ+-supportive resources.

### Youth-Specific Resources

Finally, an opportunity exists within 4-H to promote the leadership and empowerment of LGBTQ+ and allied youth. Youth participants described a need for youth-specific resources provided within the 4-H umbrella that are LGBTQ+-specific and/or LGBTQ+-supportive. They provided several examples including an LGBTQ+ club within 4-H youth groups, youth advocacy clubs for youth to learn about and practice advocacy skills, peer mentorship programs for older and younger youth to connect on shared identities and experiences, support groups, youth retreats specific to gender and sexuality, and other groups that can increase empowerment and visibility of LGBTQ+ youth.

## Discussion

Findings from this study highlight the challenges and opportunities within 4-H as identified by individuals directly connected to a 4-H program in one state. The identification of both challenges and opportunities within the organization promotes a balance of risk and strengths in considering how 4-H can better support LGBTQ+ youth. In research on rural communities and LGBTQ+ youth, Paceley (2020) suggests that this balance is an essential step toward making changes in a community; we also advocate for this balance within organizations. A wealth of literature illustrates the ways in which 4-H promotes positive development for youth broadly. This study contributes to the literature on LGBTQ+ youth positive youth development and emphasizes how 4-H can meet their commitment to diversity and inclusion by engaging with opportunities to better support the development of LGBTQ+ youth.

LGBTQ+ youth identified challenges related to safety, reliance on a gender binary, religious conservativism, and lack of diversity (Paceley et al., 2020b). Safety is a significant concern for LGBTQ+ youth across contexts (Paceley et al., 2020b), resulting in health, mental health, and educational inequities (Aragon et al., 2014; Paceley et al., 2020a; Rosario et al., 2006). Our findings add to this evidence by demonstrating how harassment and misgendering within 4-H decreases feelings of safety among LGBTQ+ youth. Importantly, research also illustrates opportunities to increase safety and promote positive development. In one study, when transgender individuals’ names and pronouns consistent with their gender identity were used, depression decreased by 71%, suicidal thoughts decreased by 34%, and suicide attempts decreased by 65% (Russell et al., 2018). Additionally, intervention by adults sends a message to all participants that harassment and misgendering are not acceptable at 4-H. The lack of intervention reported by the participants in this study is consistent with school climate research with LGBTQ+ youth, which finds there is a lack of intervention by school staff and teachers, including in rural schools, and that youth feel unsafe as a result (Palmer et al., 2012).

Challenges related to enforcement of a gender binary were present across our findings, such as practices and policies that promoted gendered activities, sleeping arrangements, and bathroom usage based on sex assigned at birth. These types of policies and practices send a message to LGBTQ+ youth, and transgender youth in particular, that they are not welcome (Paceley et al., 2020). Additionally, a recent study highlighted the experiences of LGBTQ+ youth in schools who were told they could not use bathrooms based on their gender (Porta et al., 2017). Youth were able to advocate for inclusive bathrooms with support from teachers and the school-based GSA, echoing the need for a more diverse staff to support youth in these efforts.

Research suggests that conservative ideologies are more common in rural communities (Herek, 2002; Movement Advancement Project, 2019) and are associated with greater opposition to non-heteronormative identities (Van der Toorn et al., 2017). It is not a surprise, therefore, that even though 4-H is not a religious organization, its membership and the broader community may reflect more conservative values. Alternatively, the findings from this study suggest that the 4-H guiding principles of acceptance and support for all youth provide opportunities for affirming and accepting LGBTQ+ youth and convincing those who may be resistant to do the same. This is especially important given the relationship between a supportive religious climate and reduced sexual risk-taking behaviors and substance use among LGBTQ+ youth (Hatzenbuehler et al., 2012). Finding ways to bridge conservative and LGBTQ+-supportive ideologies could be critical to the health of LGBTQ+ youth.

Our findings suggest the need for more visible and out LGBTQ+ youth and adults within 4-H—to provide mutual support, but also to promote visibility and inclusive policies and programs. Related studies have navigated the complex tensions between visibility and safety. For example, transgender youth in a rural Midwestern state identified the need for more visible transgender youth and adults in their communities, but wrestled with the safety issues inherent for more visible transgender community members (Paceley et al., 2020). In attempts to promote visibility of LGBTQ+ youth, volunteers, and staff, attention must be given to balancing the need for safety for LGBTQ+ people, as well, within both 4-H organizations and the broader community context. Safety, at minimum, must be considered simultaneous to visibility.

Importantly, the findings from this study also include strengths of 4-H and opportunities for growth. Given the inclusive mission of 4-H, incorporating LGBTQ+ identities into anti-bullying, inclusion, and non-discrimination policies is an opportunity to explicitly stand with and affirm LGBTQ+ communities. Consistent with school-based research, when a school policy does not explicitly include diverse genders and sexualities, youth remain discriminated against and victimized (Palmer et al., 2012). One type of policy the groups talked about in this study was that of dress codes. Youth described the dress code as problematic in both its language and enforcement. For example, dress code policies described formal attire in a gendered manner (suit and tie for boys, blouses for girls) and gendered bathing suit requirements.

Another opportunity exists for 4-H to connect with LGBTQ+ community organizations and groups and/or host their own LGBTQ+-specific youth groups. Pride and other community LGBTQ+ events are a critical component of LGBTQ+ youth feeling supported and affirmed in their communities (Paceley, 2016; Paceley et al., 2020); having 4-H connected to these events would go a long way toward supporting LGBTQ+ youth. Additionally, 4-H could create their own LGBTQ+-affinity group, similar to a school-based GSA, to provide support and resources (Palmer et al., 2012).

### Implications

This study has important implications for the 4-H program, rural community practice, and research. First, 4-H should consider strategies related to education, programs and policies, hiring, and community partnerships in order to increase inclusion of the LGBTQ+ community and improve the climate for LGBTQ+ youth. These strategies may also improve the climate for LGBTQ+ families, volunteers, and staff who are part of the vital operation of the 4-H program. One important strategy is education. Our findings emphasize the need for education pertaining to gender identity and sexual orientation among staff, volunteers, and youth. Education at all of these levels will not only provide resources to 4-H participants, but can help improve organizational climate, inform policies and procedures, scrutinize existing partnerships that may damage inclusion efforts, help develop new partnerships, and empower youth to create new spaces and experiences for LGBTQ+ 4-H young people. Education can be weaved into traditional training for staff and volunteers, reviewing forms and other written documents for bias, amplifying voices of LGBTQ+ 4-H members, and promoting LGBTQ+ inclusion in publications and advertisements. One resource for education and training for 4-H is respective university resources, such as offices of equity and diversity and LGBTQ+-specific groups and clubs that can inform and support inclusion efforts. Whereas geographic location can be a barrier to local, county-based, 4-H programs, local staff are capable of directing inquiries to respective resources.

4-H programs can also adapt policy and program changes to promote inclusion of LGBTQ+ youth. Gendered activities can be altered to promote the inclusion of all participants, regardless of gender identity and expression. Additionally, practices that normalize the use of pronouns consistent with gender identity and foster respect for gender diversity among youth members and staff should be implemented or enhanced. Policies that reinforce gender binaries and stereotypes, such as dress codes, should be revised to promote inclusion of all genders. The promotion of affiliated university inclusion statements and equal opportunity clauses is not enough. 4-H codes of conduct and other behavioral contracts should include gender and sexual identity in clauses that prohibit biased-based bullying in order to proactively promote inclusivity within 4-H clubs, retreats, leadership experiences, livestock programs, and fairs. Given that research demonstrates linkages between stated policies and LGBTQ+ youth experiences of safety (Hatzenbuehler, 2016) and support (Day et al., 2019), the adoption and implementation of these policies within 4-H could create a culture shift for LGBTQ+ youth members and staff.

Education and program/policy work is important and sexual orientation and gender identity equity and inclusion requires enforcement at all levels. Specific attention should be paid to equipping 4-H participants, volunteers, and staff with strategies for responding effectively to LGBTQ+ harassment and prejudice. Youth can also be equipped with resources on how to support their peers and strategies for addressing harassment and prejudice. Volunteers and staff should be trained on policy and program requirements for inclusion of LGBTQ+ youth and held accountable to uphold those standards. Given the geographic and sociopolitical dispersion of 4-H programs around the country, 4-H leadership will have to consider how to support LGBTQ+ youth, volunteers, and staff in communities that may be more hostile toward or rejecting of LGBTQ+ people and identities

Given 4-H’s goal of achieving demographic parity in 2025, 4-H needs to consider strategies that actively recruit and retain LGBTQ+ diversity in staffing and leadership. 4-H should consider how to codify and promote state implementation of strategies outlined in the *Practices for Inclusion of Individuals of all Genders and Sexual Orientation* (AEBC, 2020), developed by their LGBTQ+ champions team. 4-H would benefit from building on individual states’ existing efforts toward inclusion, like the Ohio 4-H LGBTQ+ Youth Summit, Iowa 4-H’s LGBTQ+ champions team, and Washington 4-H’s Teen Equity and Inclusion Task Force.

Finally, 4-H programs need to examine community partnerships to determine whether partners are inclusive and learn from partners’ efforts. 4-H should not partner with organizations that promote prejudice or are intolerant of the LGBTQ+ community. Instead, 4-H should cultivate and learn from partnerships with local, county, and state organizations that promote LGBTQ+ inclusion and engage in LGBTQ+ community pride events. In order for the 4-H program to fulfill its commitment to youth voice and effectively serve its young participants it needs to promote and amplify LGBTQ+ youth voices by supporting LGBTQ+ youth-created spaces, clubs, education and leadership opportunities, training and other events.

Although this study focused on the 4-H program, the findings have broader implications for rural community programs. Youth-based programs, such as after-school programs, youth groups within churches, and general support groups should consider assessing their program inclusion of LGBTQ+ youth and identify strategies to increase inclusion and support. Including youth voice and expertise in the process of assessing and identifying strategies for improvement will be critical to ensuring that these efforts are meeting the needs of diverse LGBTQ+ youth. These organizations can also lead the way in educating the broader community to increase community-level inclusion efforts, which have been shown to benefit LGBTQ+ youth in rural spaces (Fish et al., 2019; Paceley, 2016).

Finally, this study has implications for research, particularly research that includes community-based organizations. The qualitative methods used in this study were focused first on the community engaging in a SWOT analysis and working directly with staff, volunteers, and youth. Researchers aiming to improve community organizations or systems should consider similar strategies to promote engagement and research that meet the needs of local communities. Additionally, including LGBTQ+ youth in the process of conducting and using research is important to ensure our questions, methods, and findings are relevant to the youth whose lives we aim to impact.

### Limitations

This study has several notable limitations that warrant discussion. First, the data were collected within 4-H programs in one U.S state and therefore reflect the concerns and ideas of a specific population. This potentially limits the generalizability of the findings. Additionally, the groups consisted of LGBTQ+ and LGBTQ+-supportive individuals affiliated with 4-H who were motivated to make 4-H more inclusive and affirming. Their bias may have affected their impressions of current inclusion efforts and resources. LGBTQ+ youth who may have felt unwelcome and stopped participating in 4-H programs or did not join due to a lack of inclusion may have different experiences, concerns, or suggestions. Finally, because we did not audio-record participant responses, we are not able to provide quotes in support of our findings, which would help add context to bolster the evidence. In spite of these limitations, this study has several strengths. It focuses on a topic that has received no empirical attention: LGBTQ+ youth and 4-H and centers the voices and expertise of youth and adults involved with 4-H.

### Conclusion

The findings from this study contribute to the wealth of literature on the 4-H program and its commitment to positive youth development. Still, it is among the first to center LGBTQ+ youth and staff in the 4-H program with the specific goal of increasing inclusion and support. Given the vast health disparities between LGBTQ+ youth and heterosexual and cisgender youth, it is essential to enhance understanding of how both LGBTQ+ and non-LGBTQ+ organizations can improve support and inclusion for this group of marginalized young people. This is particularly important for LGBTQ+ youth in geographic spaces without access to LGBTQ+-specific supports, such as youth in rural communities. 4-H and similar organizations have important opportunities to grow and support LGBTQ+ youth.

## Figures and Tables

**Table 1.** Groups/Participants

Group	*n*	Constituent(s)	Event	Format
Group 1	40	4-H youth	Leadership retreat	SWOT
Group 2	16	4-H youth development staff	Annual staff development conference	Question prompts
Group 3	12	4-H Extension support staff	Quarterly staff meeting	SWOT
Group 4	12	4-H youth, 4-H alumni, 4-H parents	Focus group	SWOT

**Table 2.** Challenges and Opportunities for LGBTQ+ Inclusion in 4-H

Theme	Challenges	Opportunities
Organizational climate	Harassment & bullying Lack of intervention LGBTQ+ youth feeling unsafe Lack of diversity in staff & youth Enforcement of gender binary Conservative religious tone	Mission & values rooted in acceptance Principles of safety & independence No religious affiliation Increase diversity & visibility
Policies & procedures	Lack of inclusive policies Gender binary in policies	Explicitly include LGBTQ+ in policy Collaborate with university extension
Training, education, & resources	Lack of LGBTQ+ training Lack of guidance Lack of resources	Volunteer & camp counselor training Youth trainings Trauma-informed training Language, safe spaces Inclusive forms & documents
Community engagement		Allying with LGBTQ+ organizations Engagement with university Pride events
Youth-specific resources		LGBTQ+ specific groups/events Advocacy clubs Peer mentorship Youth LGBTQ+ retreats
